# Perspectives of healthcare providers on implementation of long‐acting cabotegravir plus rilpivirine in US healthcare settings from a Hybrid III Implementation‐effectiveness study (CUSTOMIZE)

**DOI:** 10.1002/jia2.26003

**Published:** 2022-09-12

**Authors:** Maggie Czarnogorski, Cindy P. Garris, Marybeth Dalessandro, Ronald D'Amico, Toyin Nwafor, Will Williams, Deanna Merrill, YuanYuan Wang, Larissa Stassek, Michael B. Wohlfeiler, Gary I. Sinclair, Leandro A. Mena, Blair Thedinger, Jason A. Flamm, Paul Benson, William R. Spreen

**Affiliations:** ^1^ ViiV Healthcare Durham North Carolina USA; ^2^ ViiV Healthcare Collegeville Pennsylvania USA; ^3^ GSK Collegeville Pennsylvania USA; ^4^ Evidera Bethesda Maryland USA; ^5^ AIDS Healthcare Foundation Los Angeles California USA; ^6^ Prism Health North Texas Dallas Texas USA; ^7^ University of Mississippi Medical Center Jackson Mississippi USA; ^8^ KC CARE Health Center Kansas City Missouri USA; ^9^ Kaiser Permanente Sacramento Sacramento California USA; ^10^ Be Well Medical Center Berkley California USA

**Keywords:** HIV‐1, antiretroviral therapy, acceptability, appropriateness, feasibility, sustainability

## Abstract

**Introduction:**

CUSTOMIZE evaluated the implementation of long‐acting (LA) cabotegravir + rilpivirine, a novel healthcare provider–administered injectable antiretroviral therapy regimen, in diverse US healthcare settings. Findings from staff‐study participants (SSPs) through 12 months of implementation are reported.

**Methods:**

CUSTOMIZE was a phase IIIb, 12‐month, single‐arm, hybrid III implementation‐effectiveness study conducted from July 2019 to October 2020 at eight US clinics of five clinic types: private practice (*n* = 2), federally qualified health centre (*n* = 2), university (*n* = 2), AIDS Healthcare Foundation (*n* = 2) and health maintenance organization (*n* = 1). Eligible patient participants received monthly cabotegravir + rilpivirine LA injections after a 1‐month oral lead‐in. At baseline, month 4 and month 12, SSPs (*n* = 3 each per clinic), including physicians, nurses or injectors, and administrators, completed quantitative surveys and semi‐structured interviews to assess implementation outcomes (acceptability, appropriateness and feasibility of intervention measures), programme sustainability and SSP perceptions of, attitudes towards, and expectations for cabotegravir + rilpivirine LA. Month 12 data collection occurred during the COVID‐19 pandemic.

**Results:**

In surveys, SSPs reported high mean total scores for acceptability, appropriateness and feasibility of cabotegravir + rilpivirine LA implementation at baseline (4.43, 4.52 and 4.38 of 5, respectively) and month 12 (4.45, 4.61 and 4.46 of 5, respectively), regardless of clinic type. At month 12, SSPs were positive about the implementation sustainability (mean Program Sustainability Assessment Tool score, 5.83 out of 7). At baseline, SSPs’ top concern was patients’ ability to maintain monthly appointments (81%); at month 12, 39% had this concern. The proportion of SSPs reporting patient injection pain or soreness as a barrier was consistent at month 12 versus baseline (48% vs. 46%). Most (78%) SSPs reported optimal implementation of cabotegravir + rilpivirine LA in their clinics was achieved in 1–3 months. In interviews, SSP‐reported strategies for successful implementation included teamwork, using a web‐based treatment planner and having a designated person to track appointment scheduling. In month 12 interviews, SSP‐reported structural changes needed for implementation included changing clinic hours and purchasing refrigerators.

**Conclusions:**

In CUSTOMIZE, cabotegravir + rilpivirine LA was successfully implemented across a range of US healthcare settings. Barriers were mitigated with minor process adjustments.

## 1 INTRODUCTION

The first complete long‐acting (LA) injectable regimen of cabotegravir and rilpivirine is recommended by treatment guidelines for the maintenance of virologic suppression in people living with HIV‐1 (PLHIV) [1, 2]. Phase III studies in virologically suppressed adults with HIV‐1 infection showed non‐inferiority of intramuscular cabotegravir + rilpivirine LA administered every 4 weeks to daily oral antiretroviral therapy (ART) at 48 weeks [3, 4]. The phase IIIb ATLAS‐2M study demonstrated non‐inferiority of cabotegravir + rilpivirine LA administered every 8 weeks to every 4 weeks dosing at 48 and 96 weeks [5, 6].

Once‐monthly and every‐2‐month cabotegravir + rilpivirine LA regimens provide less‐frequent dosing options for PLHIV compared with a daily oral pill. However, LA injections require that PLHIV attend regular clinic visits and may require additional resources and logistical changes in a clinical setting. To successfully and sustainably implement a clinic‐based treatment, such as cabotegravir + rilpivirine LA, it is important to optimize administration from the perspectives of PLHIV and healthcare staff [7]. Thus, an implementation trial was conducted to understand the level of clinic training and support needed to effectively deliver this novel regimen. Here, we report the perspectives of healthcare providers from a variety of US healthcare settings on the implementation of once‐monthly cabotegravir + rilpivirine LA into routine clinical care.

## 2 METHODS

### 2.1 Study design

CUSTOMIZE was a phase IIIb, 12‐month, single‐arm, hybrid III implementation‐effectiveness study that evaluated the acceptability, appropriateness, feasibility and sustainability of cabotegravir + rilpivirine LA implementation in clinical practice (NCT04001803). CUSTOMIZE was conducted from 2019 to 2020 in five US clinic types: private practice (*n* = 2), federally qualified health centre (FQHC; *n* = 2), university (*n* = 2), AIDS Healthcare Foundation (AHF; *n* = 1) and a health maintenance organization (HMO; *n* = 1; Table 1). One additional FQHC site withdrew after baseline staff‐study participant (SSP) interviews were completed but before enrolling any patient participants. Clinics in high HIV prevalence areas that had not previously administered cabotegravir + rilpivirine LA were screened based on their ability to perform study assessments within International Conference on Harmonization and Good Clinical Practice guidelines. Sites were chosen to create a representative sample of different clinic types, demographics and geographic regions. Of the eight participating clinics, four (50%) were located in the Southeastern United States and eight (100%) were located in jurisdictions with a high prevalence of HIV diagnoses that were prioritized in Phase I of the Ending the HIV Epidemic initiative [8]. Month 4 data collection ended in January 2020 (before the detection of COVID‐19 in the United States). Month 12 data collection was completed in October 2020 (during the COVID‐19 pandemic).

**Table 1:** Number of participating study‐staff participants at each time point

Location	Clinic type	Baseline	Month 4	Month 12
Atlanta, GA	Private practice	3	3	2^a^
Detroit, MI	Private practice	3	3	3
	Subtotal	6	6	5
Dallas, TX	FQHC	3	3	3
Kansas City, MO	FQHC	3	3	3
Washington, DC	FQHC	2^b^	0	0
	Subtotal	8	6	6
Jackson, MS	University	3	3	3
Jacksonville, FL	University	3	3^c^	3^d^
	Subtotal	6	6	6
Miami, FL	AHF	3	3	3
Sacramento, CA	HMO	3	3	3
Total for all clinics		26	24	23

Abbreviations: AHF, AIDS Healthcare Foundation; FQHC, federally qualified health centre; HMO, health maintenance organization.

^a^ The Atlanta site office administrator left between months 4 and 12 and was not replaced. Their responsibilities were assumed by the Atlanta clinic's injector.

^b^ The Washington, DC, site withdrew from the study before enrolling any patient participants. Two SSPs from this site completed baseline surveys and interviews but did not participate in month 4 or 12 activities.

^c^ The physician designated as the principal investigator at the Jacksonville site changed just before month 4 SSP surveys and interviews. However, the new principal investigator was previously involved in the study and, therefore, was asked to complete the month 4 study activities.

^d^ The staff member designated as the administrator at the Jacksonville site changed between month 4 and 12 activities. The newly designated office administrator at this site was previously involved in the study.

SSPs included lead investigators or HIV‐1 care providers (i.e. physicians), nurses or other staff administering injections (injectors) and office administrators or clinic managers (*n* = 1 each per clinic). SSPs were selected at the clinics’ discretion using a provided list that outlined key responsibilities for each role. Selected SSPs were expected to be the primary clinic staff involved with study procedures at each site. Each SSP completed surveys and interviews at baseline (*N* = 26), month 4 (*N* = 24) and month 12 (*N* = 23).

Informed consent was obtained from SSPs before any study procedures. The study was conducted in accordance with the International Conference on Harmonization Good Clinical Practice and the Declaration of Helsinki. The study protocol was approved by a central Institutional Review Board and/or local Institutional Review Boards where required.

### 2.2 Clinical intervention

After a 1‐month lead‐in with oral cabotegravir 30 mg + rilpivirine 25 mg to assess initial tolerability, patient participants received a cabotegravir 600 mg + rilpivirine 900 mg LA loading dose at month 1 and monthly cabotegravir 400 mg + rilpivirine 600 mg LA maintenance doses thereafter. Injections were scheduled on an individual patient participant basis with a ±7‐day dosing window around the target visit date, which was dictated by the date of the month 1 injection.

### 2.3 Implementation intervention

The primary implementation strategy comprised facilitation plus implementation toolkits. Implementation strategies were evaluated by clinic type and the impact on implementation outcomes was assessed. Facilitation included monthly phone meetings with representatives from each site, occurring from the beginning of patient enrolment through the last month 6 visit, to share best practices, discuss support tools and alternative workflows and identify barriers to and facilitators of successful delivery of cabotegravir + rilpivirine LA.

The toolkit contained both SSP and patient participant materials supporting education, adherence to monthly visits and clinic logistic planning. These materials were provided in digital and hard‐copy formats and through in‐person meetings. Toolkit materials were available to use at the clinics’ discretion and included a cabotegravir + rilpivirine LA fact sheet, cabotegravir + rilpivirine LA injection training video, web‐based treatment planner to assist staff in managing patient workflow, patient reminder system, hot/cold packs for patients, what‐to‐expect educational injection flash card for patients, frequently asked questions educational document for patients and a what‐to‐expect video for patients.

### 2.4 Objectives and assessments

Proctor Framework was used to evaluate the acceptability, appropriateness and feasibility of delivering cabotegravir + rilpivirine LA across clinic types [9]. Other objectives included evaluation of organizational facilitators and barriers, implementation sustainability, and safety and efficacy of cabotegravir + rilpivirine LA.

Implementation outcomes were assessed through acceptability of intervention measure (AIM), intervention appropriateness measure (IAM) and feasibility of implementation measure (FIM) surveys at baseline, month 4 and month 12 [10]. Each was a 4‐item survey that utilized a 5‐point rating scale (1 = completely disagree to 5 = completely agree) and has been previously validated under the Proctor Framework [10]. The primary endpoint was changed from baseline in AIM, IAM and FIM at month 12.

Programme sustainability was assessed in month 12 using the Program Sustainability Assessment Tool (PSAT) [11]. For the purposes of this study, two of the PSAT domains in the original scale—partnerships and funding stability—were excluded from the survey. Six domains were included: environmental support, organizational capacity, programme evaluation, programme adaptation, communications and strategic planning. Each domain was measured with five items (30 items total). Each item was assessed with an 8‐point rating scale (1 = to no extent at all and 7 = to a very great extent), as well as a not applicable/not able to answer option.

Barriers to implementation were assessed in a 23‐item survey (baseline, month 4 and month 12) that utilized a 5‐point rating scale (1 = completely disagree to 5 = completely agree; higher scores were more positive). Additional survey questions assessed SSP perceptions of the utility of the toolkit, time spent and acceptability of time spent in the clinic for injection visits, time to optimal implementation and attitudes towards and expectations for cabotegravir + rilpivirine LA. Surveys were self‐administered, and results were scanned or faxed to Evidera (Bethesda, MD, USA) for analysis.

**Figure 1:**
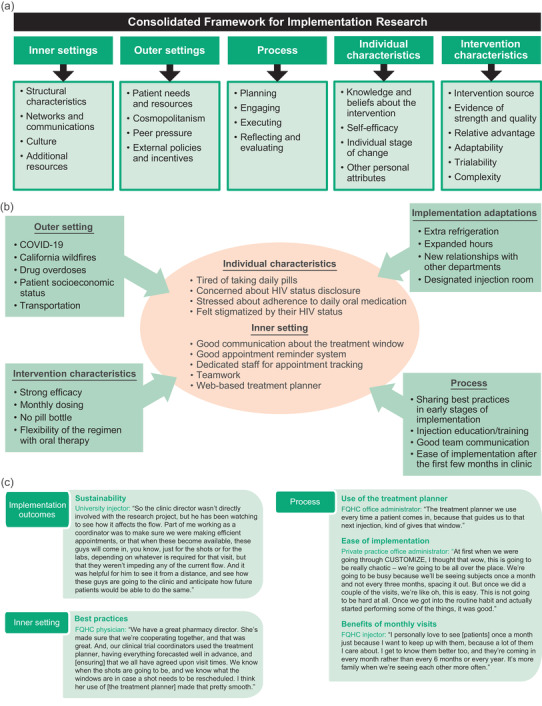
(a) Domains and constructs of the consolidated framework for implementation research. (b) Implementation results from the CUSTOMIZE study by consolidated framework for implementation research domain. (c) Staff‐study participants in their own words. Abbreviation: FQHC, federally qualified health centre.

The Consolidated Framework for Implementation Research (CFIR) provides a theoretical model for evaluating cabotegravir + rilpivirine LA administration in the clinic setting [12, 13]. The CFIR is composed of five major domains affecting implementation success: inner setting, outer setting, process, individual characteristics and intervention characteristics (Figure 1a,b) [12]. The CFIR model supports identifying barriers to and facilitators of meeting implementation goals. Semi‐structured, CFIR‐guided interviews were conducted by an interviewer from Evidera at baseline, month 4 and month 12 and addressed topics including barriers to implementation, infrastructure changes, use of toolkit resources and best practices. Baseline interviews were conducted in person; month 4 and month 12 interviews were conducted by phone. Qualitative interviews were recorded, transcribed and analysed for trends using ATLAS.ti software (version 8.1; ATLAS.ti Scientific Software Development GmbH, Berlin, Germany).

### 2.5 Statistical methods

The study size was based on estimates for the number of patient participants required to initiate potential changes in clinic flow and logistics. The sample size for SSPs was determined by the feasibility of enrolling an adequate number of clinics to obtain multiple staff members’ perspectives on implementation and to achieve thematic saturation in SSP interviews. Nine sites (*n* = 3 SSPs per site) and 135 patient participants (*n* = 15 per site) were planned for enrolment to provide adequate precision for estimations of the primary endpoint; only 115 patient participants were enrolled, in part due to the withdrawal of one FQHC site. Descriptive statistics are reported for clinic and SSP characteristics and survey measures.

## 3 RESULTS

### 3.1 Implementation outcomes

#### 3.1.1 Acceptability, appropriateness and feasibility of an intervention

**Figure 2:**
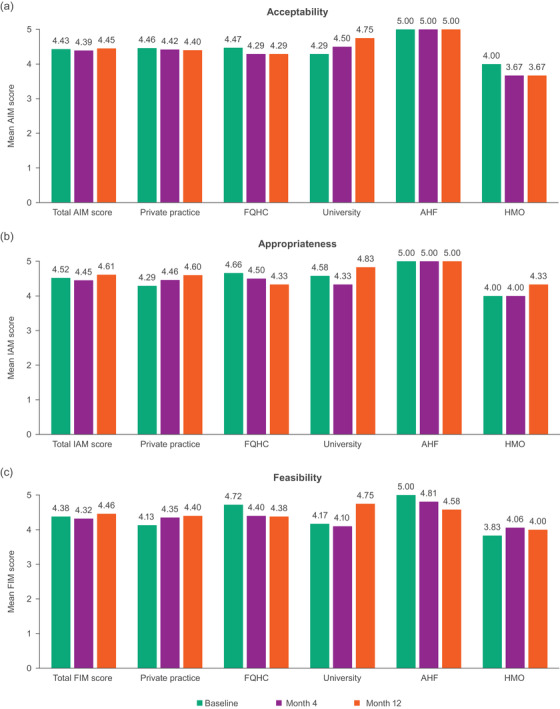
Mean total scores and mean scores for each clinic type from (a) AIM, (b) IAM and (c) FIM questionnaires over time. Each was a 4‐item survey that utilized a 5‐point rating scale (1 = completely disagree to 5 = completely agree; higher scores were more positive). Items in the AIM survey were as follows: (1) cabotegravir + rilpivirine LA meets my approval, (2) cabotegravir + rilpivirine LA is appealing to me, (3) I like the idea of cabotegravir + rilpivirine LA and (4) I welcome cabotegravir + rilpivirine LA. Items in the IAM survey were as follows: (1) cabotegravir + rilpivirine LA seems fitting, (2) cabotegravir + rilpivirine LA seems suitable, (3) cabotegravir + rilpivirine LA seems applicable and (4) cabotegravir + rilpivirine LA seems like a good match. Items in the FIM survey were as follows: (1) cabotegravir + rilpivirine LA seems implementable, (2) cabotegravir + rilpivirine LA seems possible, (3) cabotegravir + rilpivirine LA seems doable and (4) cabotegravir + rilpivirine LA seems easy to administer. Abbreviations: AHF, AIDS Healthcare Foundation; AIM, acceptability of intervention measure; CAB, cabotegravir; FIM, feasibility of implementation measure; FQHC, federally qualified health centre; HMO, health maintenance organization; IAM, intervention appropriateness measure; LA, long‐acting; RPV, rilpivirine.

High acceptability, appropriateness and feasibility of cabotegravir + rilpivirine LA were reported among SSPs at baseline, month 4 and month 12, regardless of clinic type (Figure 2). For acceptability, mean total AIM scores were consistent between baseline (4.43) and month 12 (4.45; Figure 2a). At month 12, the majority of SSPs at each site agreed or completely agreed with each of the four items, with only one SSP reporting a neutral response for item 1 (office administrator from the HMO site) and one SSP reporting a neutral response for items 2 through 4 (nurse/injector from the HMO site). For appropriateness, mean total IAM scores were 4.52 at baseline and increased at month 12 (4.61), with all SSPs at each site agreeing or completely agreeing with all four items at month 12 (Figure 2b). For feasibility, the mean total FIM scores were 4.38 at baseline and increased at month 12 (4.46; Figure 2c). At month 12, most SSPs at each site agreed or completely agreed with each of the four items, with the office administrator from a private practice reporting a neutral response for items 1 through 3 and one physician/principal investigator and one office administrator from either a private practice or the AHF site reporting a neutral response for item 4.

#### 3.1.2 Sustainability of intervention

At month 12, SSPs were positive about the sustainability of cabotegravir + rilpivirine LA implementation, with individual mean PSAT domain scores ≥5.50 (maximum score of 7) and an overall mean score of 5.83 across all domains (see Figure 1c for SSP quotations). Each PSAT domain represents concepts that are important for programme sustainability. SSPs indicated they were most confident about the communication plan for their clinics and least confident about strategic planning (mean domain score of 6.09 and 5.50, respectively). Other mean domain scores were 5.74, 5.82, 5.83 and 5.98 for organization capacity, environmental support, programme evaluation and programme adaptation, respectively.

#### 3.1.3 Medication regimen preferences

In month 12 surveys, 8 (57%) of 14 SSPs who indicated they prescribe medications in their role reported having no preference between providing cabotegravir + rilpivirine LA or prescribing or recommending daily oral tablets, two (14%) preferred cabotegravir + rilpivirine LA, one (7%) preferred daily oral tablets and three were undecided. Both individuals who preferred LA injections were nurses or injectors from either a private or university practice.

#### 3.1.4 Barriers to implementation

**Figure 3:**
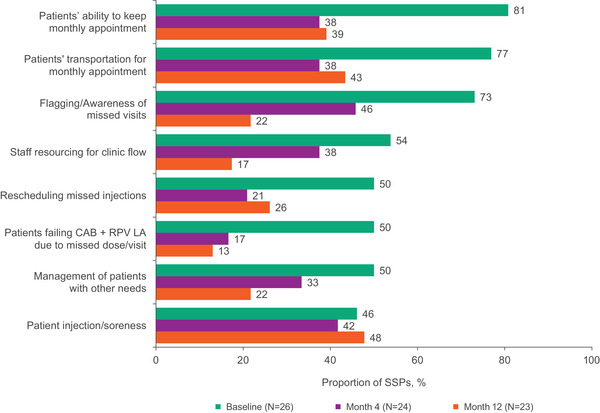
Most commonly endorsed barriers to implementation among SSPs at baseline, month 4 and month 12. Bars show the proportion of SSPs who agreed or completely agreed that each item was a barrier. Abbreviations: CAB, cabotegravir; LA, long‐acting; RPV, rilpivirine; SSP, staff‐study participant.

The proportion of SSPs indicating patient and clinic factors were potential barriers to implementation numerically decreased from baseline to month 4 (Figure 3). At month 12, the proportion indicating barriers was lower than at baseline except for patient injection pain or soreness, which was consistent between month 12 versus baseline (48% vs. 46%). At baseline, patients’ ability to maintain monthly appointments was the top‐reported concern among SSPs (81%) but was lower at month 12 (39%).

### 3.2 Implementation determinants (CFIR–informed)

#### 3.2.1 Inner settings: available resources

In month 4 interviews, nine SSPs (38%) reported that structural changes and resources were needed to accommodate cabotegravir + rilpivirine LA (Table 2). The most reported structural change was purchasing ≥1 extra refrigerator by three FQHC sites and two private practices (*n* = 1 from each clinic; 21%). In month 12 interviews, 30% of SSPs (*n* = 1 from university and *n* = 2 each from FQHC and private practice; *n* = 4 injectors and *n* = 3 physicians) reported needing to make changes for implementation, including changing clinic hours to accommodate early morning or late evening injection visits, purchasing refrigerators, designating more space for injections and increasing coordination with other departments (i.e. pharmacy). The majority of SSPs (*n* = 15; 65%) did not think their clinic underwent significant changes to accommodate CUSTOMIZE and did not anticipate their clinic would require permanent changes after month 12.

**Table 2:** Infrastructure changes by clinic type

	Clinic type				
Infrastructure change^a^	FQHC	University	Private practice	HMO	AHF
Extending clinic hours or staff working hours	✓+	✓+	✓+	+	✓
Increased coordination with other departments (i.e. pharmacy)	✓+	✓+		✓+	✓+
Purchasing new refrigerators	✓+		✓+		
Finding room space or working around room availability	✓+				✓+
Adjusting staff working hours	✓			✓	
Use of different staff for injections		✓			
Making transportation arrangements for patient participants		✓			
Scheduling injection visits in the morning or during lunch breaks			✓		
Designating certain exam rooms for injections	+				

Abbreviations: AHF, AIDS Healthcare Foundation; FQHC, federally qualified health centre; HMO, health maintenance organization.

^a^ Reported in interviews by ≥1 staff‐study participant at any time point. “✓” indicates the factor was endorsed at month 4. “+” indicates the factor was endorsed at month 12.

In interviews, key strategies for successful implementation were good communication about the treatment window with patients, a good appointment reminder system and a designated person to track appointment scheduling and rescheduling (Figure 1c). Additional strategies were teamwork and the use of the web‐based treatment planner.

#### 3.2.2 Inner settings: readiness for implementation and implementation climate

In month 12 interviews, FQHC SSPs indicated that initial concerns about leadership support were mitigated by positive clinical data and ease of implementation. University‐based clinics’ initial concern about patients keeping appointments was mitigated through tracking and a patient‐friendly reminder system. Private practices were initially concerned about “chaos” created by the frequency and length of injection visits; by month 12, wait times were short (∼5 minutes in the waiting room and ∼10 minutes in the exam room), and increased touchpoints benefited the patient–provider relationship. In month 12 interviews, 16 SSPs (70%) commented that there were benefits to monthly clinic visits, such as more frequent sexually transmitted infections and preventative screenings and closer monitoring of diabetes and hypertension. The AHF provider reported that they may need additional exam rooms and space to accommodate patients. Their patients’ excitement about cabotegravir + rilpivirine LA was surprising to them. The HMO site reported that implementation was less challenging with more experience and that engaged staff, teamwork and clarity about scheduling were keys to success.

Best practices varied by clinic type and included checking in with patients after their first injection (FQHC), adding the “Pharmacist in Charge” to their morning huddles (AHF), using telehealth for sharing information with patients (HMO), scheduling visits >1 month in advance (university) and designating specific time slots for walk‐in injection visits for patients needing to reschedule (private practice).

#### 3.2.3 Outer settings: patient needs and resources, and external policy and incentives

COVID‐19 pandemic–related closures began in the United States in early 2020, when CUSTOMIZE was about halfway completed. Although major changes to clinic operations and hospital policies occurred to ensure patients were adherent to COVID‐19 precautions and restrictions, all clinics were able to continue providing cabotegravir + rilpivirine LA. Changes made by clinics during the COVID‐19 pandemic included implementing symptom checks and telephone screening in advance to shorten appointments, expanding waiting rooms to maintain social distancing, avoiding waiting rooms by having patients call the clinic upon arrival, having staff available to assist with scheduling changes and implementing telehealth visits when feasible. Staff noted keys to success included having a plan to deliver oral ART medications to patients unable to attend an injection visit for COVID‐19–related reasons. All (100%) SSPs reported patients remained willing to attend clinic appointments during the pandemic. The AHF site was temporarily closed due to COVID‐19 but was able to reschedule their patients to receive LA injections within the treatment window; all other sites continued operating without closures. Despite eight patients needing short‐term oral therapy with cabotegravir + rilpivirine tablets to cover missed injections for COVID‐19–related reasons, all clinics maintained their patients on continuous ART throughout the study and all patients remained virologically suppressed [14]. No SSPs reported any patient participant requested a return to oral ART because of the pandemic.

In interviews at baseline and month 4, SSPs reported many external factors that may influence cabotegravir + rilpivirine LA implementation, including treatment cost or insurance reimbursement issues, transportation issues and patient socio‐economic status (Table 3). By month 12, treatment cost or insurance reimbursement issues was the only external factor reported.

**Table 3:** SSP‐perceived external factors affecting cabotegravir + rilpivirine LA implementation by clinic type

	Clinic type				
External factor^a^	FQHC	University	Private practice	HMO	AHF
Patient socio‐economic status	●✓	●✓			
Living conditions of PLHIV^b^	✓			✓	
Being able to take time off work^b^	✓			✓	
Transportation issues^b^	●✓	✓	●✓	✓	✓
Comorbid health conditions	✓	✓		✓	
Rural versus urban geography		✓			
Stigma of being seen at clinics		●✓			✓
Treatment cost/insurance reimbursement issues	●✓+	●✓+	●✓+	●✓+	

Abbreviations: AHF, AIDS Healthcare Foundation; FQHC, federally qualified health centre; HMO, health maintenance organization; PLHIV, people living with HIV.

^a^ Reported in interviews by ≥1 staff‐study participant at any time point. “●” indicates the factor was endorsed at baseline. “✓” indicates the factor was endorsed at month 4. “+” indicates the factor was endorsed at month 12.

^b^ Factors that were reported during facilitation calls.

#### 3.2.4 Process: planning and engaging

Monthly facilitation calls were conducted through month 6 to help with planning and best practice sharing. The first few calls were helpful for understanding initial obstacles at sites. By month 12 interviews, 10 (43%) SSPs said they did not need additional monthly facilitation calls and best practice sharing.

Throughout the study, ViiV Healthcare provided access to resources to support patient adherence, patient and provider education, and planning and logistical support. According to SSPs at month 12, the most frequently used resources in the CUSTOMIZE toolkit included hot and cold packs for patients, face‐to‐face injection training and video or online injection training (Table 4). The “what to expect” patient video was used more in the earlier months when sites were becoming accustomed to the toolkit resources compared with later months. The use of the treatment planner was highly valued, but scheduling practices varied by clinic type (Figure 1c). Some clinics found scheduling one monthly appointment at a time was most efficient for the clinic and patient, whereas other clinics (University and HMO) found that scheduling >1 month at a time worked best to limit overbooking. Private practices were more likely to use the “what to expect” video and treatment planner.

**Table 4:** SSPs’ ratings of most and least used toolkit items (month 4 and month 12)

Toolkit item	Month 4, % (*N* = 24)	Month 12, % (*N* = 23)
Most used toolkit items		
Hot/cold packs for patients	75	91
Face‐to‐face injection training	63	83
Video/online training on how to give a cabotegravir + rilpivirine LA injection	63	74
Facilitation group calls	79	70
What‐to‐expect factsheet for patients	63	70
Patient video of what to expect	67	61
Least used toolkit items		
Trial guide app	21	4
Patient reminder—electronic app (ViiV Healthcare provided)	25	9
FAQ chatbot	25	13
Web‐based clinic capacity planner	8	13
Web‐based health clinic capacity planning tool	8	13
Patient reminder—SMS/text (ViiV Healthcare provided)	21	22

Abbreviations: FAQ, frequently asked question; LA, long‐acting; SMS, short message service; SSP, staff‐study participant.

#### 3.2.5 Process: champions

Physicians were primarily the champions early in the process, identifying potential participants to enrol, answering patient questions about the treatment and/or trial and being responsible for decisions regarding the trial. Over time, responsibilities shifted, and the role of most physicians decreased, whereas office administrators and injectors remained involved in the logistics of scheduling and conducting injection visits. Injectors also participated in identifying patient participants, answered patient questions and handled issues regarding the logistics of trial management, such as space utilization and infrastructural requirements. Office administrators screened and consented to patients once identified by the physician or injector and were responsible for the day‐to‐day function of the trial.

#### 3.2.6 Process: executing, and reflecting and evaluating

**Figure 4:**
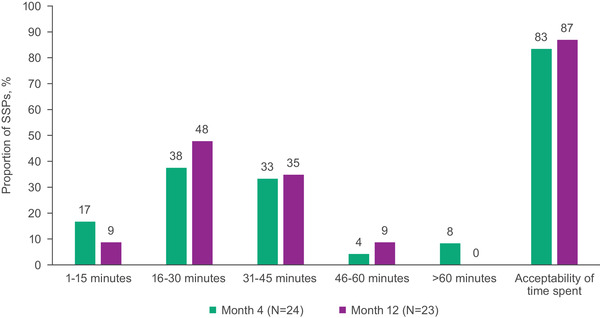
Time spent and acceptability of time spent in clinic or practice for injection visits from the perspective of SSPs. Bars for acceptability of time spent show the proportion of SSPs who reported that the time spent was very or extremely acceptable. Abbreviation: SSP, staff‐study participant.

At months 4 and 12, most SSPs reported patient injection visits lasted 16–45 minutes and the amount of time patients spent in the clinic for injection visits was very or extremely acceptable (Figure 4). The median study visit length from the start to the end of the appointment was 32 minutes at months 5 and 11. None reported it was not at all acceptable.

At month 12, most SSPs reported it took 1–3 months (78%) or 4–6 months (17%) for their clinic or practice to optimally implement cabotegravir + rilpivirine LA; none reported it took 10–12 months or responded that they were still working on it. In month 12 interviews, most SSPs indicated cabotegravir + rilpivirine LA implementation had been “easy” (*n* = 13; 57%) or “not too bad” (*n* = 7; 30%) despite initially thinking it might be “chaotic” (Figure 1c).

In month 12 interviews, SSPs described patients’ treatment adherence and treatment effectiveness as indicators of successful implementation. Additional benefits of patients coming into the clinic each month included improved patient engagement and the ability to address patients’ non‐HIV–related health concerns (Figure 1c).

#### 3.2.7 Individual characteristics: knowledge and beliefs about the intervention, individual stage of change and personal attributes

In SSP surveys, the top four characteristics of PLHIV most suited for monthly injections at baseline and month 12 included patients who were tired of taking daily pills, concerned about HIV status disclosure, stressed about adherence to daily oral medication and felt stigmatized by their HIV status (each endorsed by ≥74% of SSPs; Table 5). In month 12 interviews, SSPs reported that PLHIV who are reliable (i.e. adherent to visits and medication; *n* = 5; 22%), do not like taking pills and are younger (*n* = 2; 9%) were best suited for LA injections; two SSPs (9%) reported that all PLHIV were suitable for LA therapy.

**Table 5:** SSP‐reported characteristics of PLHIV most appropriate for cabotegravir + rilpivirine LA injections

Characteristic, %	Baseline (*N* = 26)	Month 12 (*N* = 23)
Patients who have concerns about HIV status disclosure	81	74
Patients who feel stigmatized by their HIV	77	74
Patients who are tired of taking pills daily	89	74
Patients who experience stress or anxiety over daily adherence to their oral medications	81	74
Female	69	70
Patients adherent to oral HIV medications	73	65
Male	73	65
PLHIV for 3–10 years	65	61
Younger patients (<35 years)	69	61
Older patients (>50 years)	58	61
Transgender	65	61
PLHIV for >10 years	62	57
Patients with more structured lifestyles (working regularly, stable income, stable housing and stable relationships)	62	57
All patients	27	52
Long‐term HIV survivors	50	52
Patients who are frequent travellers or have changing work/school hours	46	52
Patients with more chaotic lifestyles	50	52
Patients non‐adherent to oral HIV medications	58	48
Patients with a psychiatric comorbidity	46	44
Homeless or unstably housed patients	42	39
Newly diagnosed patients	46	30
Treatment‐naive patients	39	30
Injection drug users	50	30
Incarcerated or temporarily incarcerated patients	27	22

Abbreviations: LA, long‐acting; PLHIV, people living with HIV.

In month 4 interviews, 67% of SSPs (*n* = 16) indicated that patients’ reactions to receiving cabotegravir + rilpivirine LA were positive, reporting that patients were happy about no longer needing to take daily oral HIV medications and felt LA treatment would be simpler and fit with their life better than their daily oral regimen.

#### 3.2.8 Intervention characteristics: evidence of strength and quality, relative advantage, adaptability, trialability and complexity

In month 12 interviews, SSPs stated their confidence in cabotegravir + rilpivirine LA increased after knowing how well the medication worked for their patients. Many SSPs were surprised by the high patient interest and willingness to attend clinic visits for monthly appointments without complaint. Many SSPs noted important learnings due to the COVID‐19 pandemic and reported that temporary oral therapy was easy to implement and provided flexibility if patients could not attend an injection visit. These factors increased provider confidence in prescribing cabotegravir + rilpivirine LA to a wider variety of PLHIV.

## 4 DISCUSSION

After 12 months of once‐monthly cabotegravir + rilpivirine LA implementation in the CUSTOMIZE study, healthcare providers across diverse US clinic types and geographic locations perceived implementation as successful. Mean scores for acceptability, appropriateness and feasibility were high at baseline and either increased or remained consistent with baseline scores at month 12, despite much of the study occurring during the COVID‐19 pandemic. Healthcare providers were positive about the sustainability of implementing cabotegravir + rilpivirine LA, with high scores reported for each PSAT domain, suggesting confidence in programme sustainability based on currently employed implementation strategies. Overall, cabotegravir + rilpivirine LA implementation was successful, with high rates of adherence and satisfaction across participating sites, despite many patient participants having a lower socio‐economic status and facing many challenges, including the COVID‐19 pandemic [14].

Overall, implementation of monthly cabotegravir + rilpivirine LA exceeded SSP expectations, and perceived barriers to implementation decreased from baseline to month 12. Barriers were mitigated with minor process adjustments that varied by clinic type, with most SSPs reporting optimal implementation occurred within the first 3 months and did not require significant changes in their clinics. Teamwork among clinic staff, ongoing communication with patients and the use of an online treatment planner facilitated successful implementation.

Our findings provide initial answers to questions that have been raised about the initial steps for implementing LA ART in real‐world settings [7]. For example, SSPs identified key characteristics of patients appropriate for LA therapy, logistical requirements for implementing monthly injections, patient management strategies and approaches to staffing and staff training. Healthcare providers were pleasantly surprised at their patients’ enthusiasm for this new regimen, despite the requirement for monthly office visits. Because the every‐2‐month regimen will require similar strategies for successful implementation, the findings from this study will likely be applicable to that dosing regimen. Although CUSTOMIZE provided useful information for the initial implementation of cabotegravir + rilpivirine LA before regulatory approval, future real‐world analyses are needed to assess the impact of real‐world factors, such as insurance coverage and appointment scheduling once the patient population increases. For example, filing preapproval letters and claims with insurance companies will likely result in a time and paperwork burden among clinic staff [15].

This study has some limitations. Survey results, including differences between clinic types, should be cautiously interpreted because of the small number of SSPs and sites for each clinic type included in the study. The SSPs were not the same across time points at some sites due to staffing changes. Despite this small sample size, CUSTOMIZE enrolled a racially and ethnically diverse patient population from diverse sites and geographic locations, which were generally representative of different types of HIV‐1 care clinics and the US population of PLHIV [14, 16]. The number of patient participants enrolled per site was determined as the estimated number of patient participants that could necessitate changes in clinic practice for initial implementation; additional implementation strategies for scale‐up may be identified when a larger population of PLHIV receive LA ART at any one clinic. CUSTOMIZE was conducted before regulatory approval of cabotegravir + rilpivirine LA; therefore, some factors were non–real‐world (i.e. medications were provided, so no reimbursement or insurance processing was evaluated). However, unanticipated real‐world challenges, including the COVID‐19 pandemic, had minimal impact.

## 5 CONCLUSIONS

Overall, the results from the CUSTOMIZE study demonstrate that cabotegravir + rilpivirine LA was successfully implemented across a range of US healthcare settings with minor adjustments to clinic logistics and infrastructure, thus providing important insights into cabotegravir + rilpivirine LA implementation in post‐approval, real‐world settings.

## COMPETING INTERESTS

MC, CG, MD, RD, TW, DM and WS are employees of ViiV Healthcare and may own stock in GSK. WW and YW are employees of and may own stock in GSK. LS is an employee of Evidera, which receives funding from ViiV Healthcare for their work. MW serves on the Janssen HIV Prophylactic Vaccine Advisory Board. GS reports grants and personal fees from Janssen, ViiV Healthcare and Merck, and grants from Gilead Sciences. LM reports grants and personal fees from Gilead Sciences, GSK, ViiV Healthcare and MSD, and grants from Janssen, Visby Medical, ThaiMed, Evofem Biosciences, SpeeDx Pty Ltd and Lupin Pharmaceutical. BT owns stock in ViiV Healthcare. JF has served as a principal investigator for ViiV Healthcare. PB has nothing to disclose.

## AUTHORS’ CONTRIBUTIONS

MC, CPG, RD, LS and WRS contributed to the conception of the study. MC, CPG, MD, RD, TN, WW, DM, YW, LS and WRS contributed to the design of the study. WW, DM, LS, MBW, GIS, LAM, BT, JAF and PB contributed to the acquisition of data. MC, CPG, MD, RD, TN, YW and LS contributed to the analysis and interpretation of data. MC, CPG and LS contributed to drafting the manuscript. All authors contributed to critically revising the manuscript for important intellectual content and approve the manuscript for publication.

## FUNDING

This study was funded by ViiV Healthcare.

## Data Availability

Anonymized individual participant data and study documents can be requested for further research from www.clinicalstudydatarequest.com.
